# Wheat Grain Filling Is Limited by Grain Filling Capacity rather than the Duration of Flag Leaf Photosynthesis: A Case Study Using *NAM* RNAi Plants

**DOI:** 10.1371/journal.pone.0134947

**Published:** 2015-08-04

**Authors:** Philippa Borrill, Brendan Fahy, Alison M. Smith, Cristobal Uauy

**Affiliations:** 1 John Innes Centre, Norwich Research Park, Norwich, United Kingdom; 2 National Institute of Agricultural Botany, Cambridge, United Kingdom; Institute for Sustainable Agriculture (IAS-CSIC), SPAIN

## Abstract

It has been proposed that delayed leaf senescence can extend grain filling duration and thus increase yields in cereal crops. We found that wheat (*Triticum aestivum*) *NAM* RNAi plants with delayed senescence carried out 40% more flag leaf photosynthesis after anthesis than control plants, but had the same rate and duration of starch accumulation during grain filling and the same final grain weight. The additional photosynthate available in *NAM* RNAi plants was in part stored as fructans in the stems, whereas stem fructans were remobilised during grain filling in control plants. In both genotypes, activity of starch synthase was limiting for starch synthesis in the later stages of grain filling. We suggest that in order to realise the potential yield gains offered by delayed leaf senescence, this trait should be combined with increased grain filling capacity.

## Introduction

In wheat breeding it has been widely assumed that extending green canopy duration by delaying senescence will extend the grain filling period and increase grain yield [1]. Indeed positive correlations have been reported between grain yield and the duration of flag leaf photosynthesis in wheat [2, 3]. However, as for other cereal crops such as sorghum [4, 5], these advantages of extended green canopy duration are usually apparent only in stress environments [6]. For example across 463 wheat lines from the International Maize and Wheat Improvement Centre (CIMMYT) there was a correlation between yield and a prolonged period of flag leaf photosynthesis only under drought and/or heat stress [7]. Similarly, a prolonged period of wheat flag leaf photosynthesis may be correlated with increased yield under low nitrogen conditions, but under high nitrogen either no correlation or a negative correlation has been reported [8, 9].

The lack of effect of prolonged photosynthesis on grain yield in optimal conditions may reflect limitation of grain yield by sink rather than source capacity in these conditions. Numerous studies have concluded that wheat grain growth is limited by sink capacity [10, 11, 12], and this idea was recently supported by the absence of any significant relationships between photosynthetic parameters and grain yield in elite wheat varieties in the UK [13]. Other studies, however, report that relatively minor reductions in leaf photosynthetic capacity can adversely affect grain yield [14, 15]. In general, it has proved difficult to devise direct and unambiguous means of uncovering the relative importance of source and sink capacity in determining yield in wheat.

Most studies of source-sink relationships have used invasive physical manipulations of source or sink capacity, for example by grain removal or flag leaf removal or shading [10, 11, 16]. The flag leaf has often been the target of manipulations because it is the leaf which contributes the largest proportion of photosynthate to grain filling [17, 18]. The effects of these manipulations have generally been assessed simply in terms of final grain yield, rather than a full analysis of grain filling. Another important contributor to grain filling which is frequently overlooked in source-sink studies is fructan, the major storage carbohydrate in the vegetative tissues of wheat. Fructan accumulates in stem internodes and leaf sheaths, and is normally remobilised during the later stages of grain filling [19]. The fructan pool is thought to accommodate “excess” photosynthate when carbon assimilation exceeds the demand for carbon for grain filling. The size of the pool therefore gives an indication of the balance between sink and source activity in the plant. The comparison of plants with intrinsically different times of flag leaf senescence, with respect to both the decline in flag leaf photosynthetic capacity and the rate of and capacity for grain filling represents a less disruptive and potentially more informative way to study source-sink relationships than physical manipulations. In practice this has proved challenging. Several studies have compared varieties in which the timing of both senescence and flowering is different [20, 21], confounding the specific relationship between senescence and yield. Isogenic lines which flower at the same time usually show only small differences in the onset of senescence [22], making effects on grain filling difficult to detect.

Our previous work provides a valuable new tool for studying source-sink relationships in wheat. We have developed transgenic lines of wheat (referred to as *NAM* RNAi lines) with reduced expression of the homologues of the *NAM-B1* transcription factor (*NAM*-*A1*, *D1*, *B2*, and *D2*) [23]. Grains of these lines have 30% less protein, iron and zinc than grains of control plants. Importantly, they also have normal rates of pre-anthesis development but strong delays in flag leaf senescence after anthesis. Grain weight and yield is reported to be similar to that of control plants under both optimal and stress conditions [23, 24], but the relationship between flag leaf photosynthesis, the timing of flag leaf senescence, and grain filling has not been examined.

Our aim in this study was to shed new light on the relationship between flag leaf photosynthesis and grain filling, by exploiting the strongly-delayed senescence of the *NAM* RNAi lines. Here, we report that flag leaves of *NAM* RNAi plants are photosynthetically active for ten days longer than those of wild-type (control) plants, but although this additional photosynthesis occurs during grain filling the grains of the two genotypes accumulate starch at the same rate and reach maturity at a similar time. In both genotypes, starch accumulation appears to be limited by the activity of starch synthase. Throughout grain filling, *NAM* RNAi plants retain more storage carbohydrate in the form of fructan in their stems than control plants. Taken together these results suggest that grain filling is independent of the timing of senescence of the rest of the plant. They cast doubt on whether yield can be increased by breeding strategies aimed at delaying flag leaf senescence alone. Better prospects are offered by an integrated approach, in which delayed senescence is coupled with an increased and prolonged capacity for grain filling.

## Materials and Methods

### Plant material and sampling

Transgenic wheat plants cv. Bobwhite (two independent events: L19 and L23) containing a RNA interference (RNAi) construct which down-regulates the expression of *NAM-B1* homologues (*NAM* RNAi) [23] and null-segregants (control) plants were used for this study. For each genotype (L19 RNAi, L19 control, L23 RNAi and L23 control), 55 grain were sown into P40 trays with 85% fine peat, 15% horticultural grit; containing 2.7 kg m^-^³ Osmocote (3–4 months longevity), 0.5 kg m^-^³ wetting agent H_2_Gro (Everris), 4 kg m^-^³ Maglime (Francis Flower) and 1 kg m^-^³ PG Mix fertilizer (www.yara.co.uk). Plants were potted on at 2–3 leaf stage to 1L pots with 1 plant per pot in 40% medium grade peat, 40% sterilized soil, 20% horticultural grit; containing 1.3 kg m^-^³ PG Mix 14-16-18 (www.yara.co.uk), 1 kg m^-^³ Osmocote Exact Mini, 0.5 kg m^-^³ wetting agent H_2_Gro, 3 kg m^-^³ Maglime and 300 g m^-^³ Exemptor (Bayer). Transgenic and corresponding control lines were grown together in the same controlled environment room with 16 h light (300 μmol m^-2^ s^-1^) at 20°C and 8 h dark at 15°C and constant 70% humidity. Genotypes were verified by PCR for presence/absence of the RNAi construct (primers table S5 [23]). The main spike was tagged at anthesis on each plant. Only the main tiller was used for experiments, with the main tiller of each plant constituting an individual biological replicate. Plants were randomly assigned to different experiments and time points. The number of replicates used is indicated in each figure legend.

### Photosynthetic rate

Photosynthesis was measured under ambient conditions using a Li6400 portable photosynthesis system (LI-COR Biosciences, Lincoln, Nebraska, USA). The cuvette conditions were 400 μmol CO_2_ mol^-1^ and photosynthetically active radiation (PAR) of 300 μmol m^-2^ s^-1^. The leaf temperature was set at 20°C. Gas exchange properties were logged when the system reached a pre-determined stability point (coefficient of variation ≤ 2%), usually after ~5 min. The cuvette had an area of 6 cm^2^: where the flag leaf was <2 cm wide, its width was measured and the value used in subsequent calculations.

### Calculation of glucose produced by prolonged photosynthesis in *NAM* RNAi plants

Assumptions were: *NAM* RNAi plants had ten days more photosynthesis than control plants; rates of photosynthesis before this period and during the period of senescence were identical; during the extra ten days of photosynthesis, the rate was a constant 8.6 μmol CO_2_ m^-2^ s^-1^ (average of the 20 and 30 DAA time-points for *NAM* RNAi plants); the average flag leaf area was 0.00400 m^2^.

During the ten days with 16 h light: 8.6 μmol CO_2_ m^-2^ s^-1^ x 576,000 s x 0.00400 m^2^ = 19,814 μmol CO_2_ were assimilated per flag leaf. Assuming that photorespiration decreases the efficiency of CO_2_ fixation by 20%, 7.2 mol CO_2_ are required to make 1 mol glucose. Therefore 19,814 μmol CO_2_ is equivalent to 2,752 μmol glucose. The M_r_ of glucose is 180 therefore 495 mg more glucose equivalents were assimilated per flag leaf in *NAM* RNAi than in control plants.

### Flag leaf area estimation and chlorophyll content

Flag leaf area was estimated from measurements of the length of the blade and its width at 10 cm intervals.

Chlorophyll content was measured using a handheld chlorophyll meter (SPAD-502, Konica Minolta, Warrington, UK).

### Grain moisture content and yield components

Eight grains from the central third of the main spike were dissected out, weighed, and placed in open Eppendorf tubes at 60°C for 72 h. Dried grains were weighed, and the percentage moisture was calculated.

Ears from all tillers of 12 plants of each genotype were threshed by hand. Grain number, size and mass were measured using the MARVIN-universal grain analyser (GTA Sensorik GmbH, Neubrandenburg, Germany).

### Enzyme and metabolite assays

For enzyme assays, ten grains from the central third of the main spike were dissected out, snap-frozen in liquid nitrogen and stored at -80°C. After weighing, sufficient frozen grains to obtain a tissue:extract ratio of ~ 350 mg mL^-1^ were homogenized using a 7 mm stainless steel ball in a ball mill (QIAGEN Retsch, Valencia, USA) in 0.5 mL extraction medium [100 mM 3-(N-morpholino)propane sulfonic acid (MOPS) pH 7.2, 5 mM MgCl_2_, 5% (v/v) glycerol, 5 mM dithiothreitol (DTT), 10 mg mL^-1^ bovine serum albumin (BSA) and 1% (w/v) polyvinylpolypyrrolidone (PVPP)]. Extracts were centrifuged at 10,000 x g for 5 min at 4°C, and 100 μL aliquots of supernatant were snap frozen in liquid nitrogen.

AGPase activity was assayed using the method of Rösti *et al*. [25] adapted for microtitre plates; starch synthase activity was assayed using the Dowex method [26], measuring the incorporation of ^14^C from ADP[^14^C]glucose into glucans.

For metabolite assays harvesting and extraction of grains was as above, except that the extraction medium was 0.77 M perchloric acid. Extracts were centrifuged at 10,000 x g for 10 min at 4°C and supernatants and pellets were used for sugar and starch assays respectively. Metabolites were measured by the absorbance changes caused by enzymatic digestion according to the method of Hargreaves and ap Rees [27] adapted for microtitre plates.

### Measurement of stem fructans

Main tillers were harvested at 10 and 35 DAA. Tillers were divided into exposed peduncle, enclosed peduncle, internode 1 and internode 2; the latter three were removed from the leaf sheaths. These regions were subdivided into 2–5 mm sections, snap-frozen in liquid nitrogen and stored at -80°C.

Fructans were measured using the Megazyme fructan assay (K-Fruc 03/14, Megazyme, Bray, Ireland) according to the manufacturer’s instructions, except that all steps were scaled down for 1.2 mL deep-well plates or microtitre plates.

### Statistical tests

Statistical tests were carried out in Genstat 15^th^ Edition using Student’s t-tests between genotypes for each time-point.

## Results

### Photosynthesis is prolonged in *NAM* RNAi plants but grain weight is not increased

**Fig 1:**
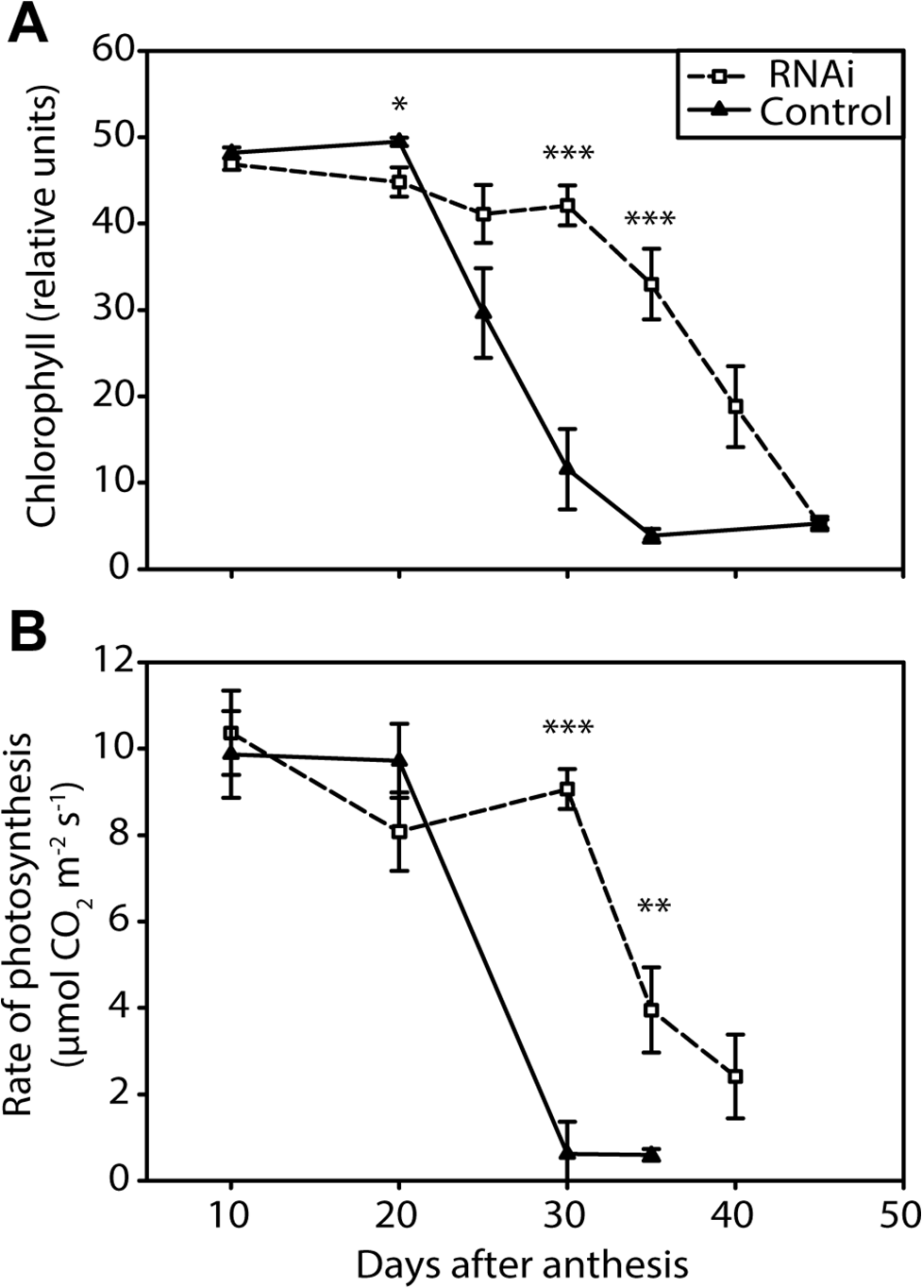
Flag leaf senescence characteristics of L19 *NAM* RNAi (open squares) and control (filled triangles) plants. (A) Chlorophyll. For each time point, each flag leaf was measured with a hand-held chlorophyll meter at ten positions across the surface. Values are means of the average measurements for flag leaves from six plants (biological replicates), ± SEM (standard error of the mean). (B) Photosynthesis. The rate of photosynthesis was measured under ambient conditions using a portable photosynthesis system. Values are means of measurements on four to six plants (biological replicates), ± SEM. Asterisks denote significant differences between genotypes at p < 0.05 (*), p < 0.01 (**) and p < 0.001 (***).

Under our growing conditions the *NAM* RNAi plants developed similarly to control plants before anthesis, reaching anthesis one day later than control plants at 53 days after sowing (p = 0.008, 47 control and 50 RNAi plants, transgenic line L19). After anthesis the *NAM* RNAi plants showed a strong delay in monocarpic senescence. Their flag leaves retained high chlorophyll levels and high rates of photosynthesis for approximately ten days longer than those of control plants (Fig 1). In control plants both chlorophyll content and the rate of photosynthesis fell from a maximum to nearly zero between 20 and 30 days after anthesis (DAA). In the *NAM* RNAi plants this fall occurred ten days later, between 30 and 40 DAA. Similar development and senescence patterns were observed in a second transgenic line (L23). L23 *NAM* RNAi plants reached anthesis 3 days later than control plants at 59 days after sowing (p < 0.001, 50 control and 49 RNAi plants). Flag leaf chlorophyll content and rate of photosynthesis were maintained at high levels for longer in L23 *NAM* RNAi than control plants (S1 Fig), similar to L19.

To assess the potential impact on yield of prolonged photosynthesis in *NAM* RNAi plants we calculated the amount of carbon assimilated during this time for the L19 transgenic line. The areas of *NAM* RNAi and control flag leaves were the same (Table 1, p = 0.91). Rates of photosynthesis up to 20 DAA and during the period of loss of photosynthetic capacity were equivalent in *NAM* RNAi and control plants. We therefore assumed that the only difference between the two genotypes was that *NAM* RNAi plants had an additional ten days of photosynthesis at a rate of 8.6 μmol CO_2_ m^-2^ s^-1^ (average of the 20 and 30 DAA time-points). Thus ~495 mg glucose equivalents were produced by CO_2_ assimilation per flag leaf in *NAM* RNAi plants during this period, assuming 20% loss of assimilated carbon through photorespiration (calculation in Materials and Methods section). Considering that both genotypes had on average 4.2 tillers (Table 1), the flag leaves of *NAM* RNAi plants produced 2,079 mg more glucose equivalents per plant than those of control plants in the post-anthesis period.

**Table 1:** Architecture and yield components of L19 *NAM* RNAi and control plants.

	Control	RNAi
Main tiller flag leaf area (cm^2^)	40.3 ± 1.6	40.0 ± 1.8
Tillers per plant	4.2 ± 0.3	4.2 ± 0.2
Grain mass per plant (g)	7.8 ± 0.3	7.3 ± 0.3
Thousand grain weight (g)	39.7 ± 0.9	39.4 ± 0.7
Grain number per plant	192 ± 11	178 ± 10
Grain area (mm^2^)	17.5 ± 0.3	17.9 ± 0.2
Grain length (mm)	6.1 ± 0.05	6.1 ± 0.03
Grain width (mm)^a^	3.5 ± 0.03	3.6 ± 0.02

Values are means of measurements on 12 individual plants (biological replicates), ± SEM (standard error of the mean). Grain measurements include grains from all tillers.

^a^ Significant difference between genotypes at p < 0.05 (*).

Other parameters were not significantly different between genotypes.

Despite this extended period of photosynthesis, and hence a greater amount of carbon assimilated, the final grain mass per *NAM* RNAi plant was not significantly different from that of control plants (Table 1, p = 0.25). Other yield components including thousand grain weight, grain number per plant, grain area and grain length were the same in control and *NAM* RNAi plants (Table 1). Mature grains of *NAM* RNAi plants were about 3% wider than those of control plants (Table 1). The second transgenic line L23 also showed no increase in grain mass per plant, thousand grain weight, grain number per plant or grain size for *NAM* RNAi plants compared to control plants (S1 Table).

To understand why grain mass did not increase despite prolonged photosynthesis we decided to study in detail the transgenic line L19.

### Grain development is independent of flag leaf senescence in *NAM* RNAi plants

**Fig 2:**
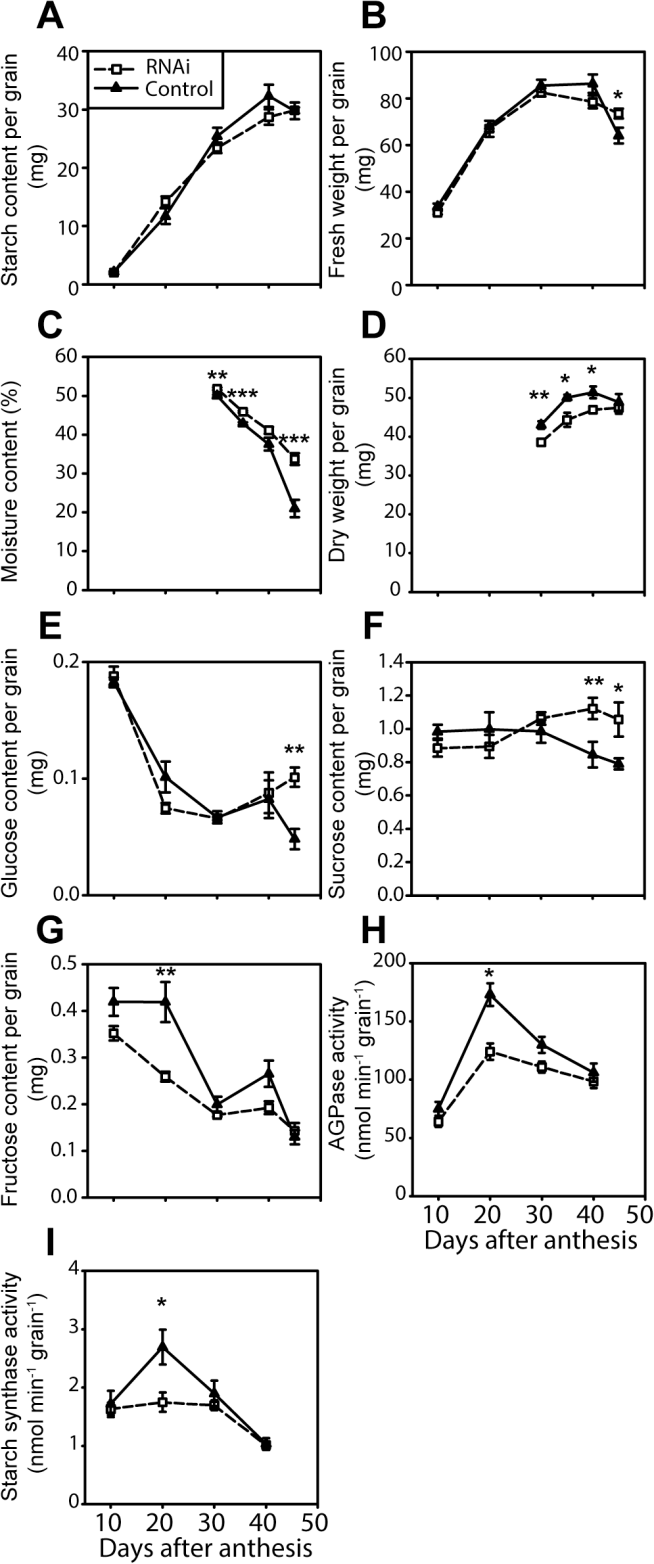
Grain filling parameters in L19 *NAM* RNAi (open squares) and control (filled triangles) plants. (A) Starch content, (B) fresh weight, (C) moisture content, (D) dry weight, (E) glucose content, (F) sucrose content, (G) fructose content, (H) AGPase activity and (I) starch synthase activity. Values are means of measurements on five or six individual plants (biological replicates), ± SEM. For each plant, measurements were on eight to ten grains from the centre of the main spike. For metabolite and enzyme assays, these grains were pooled to make a single extract per plant. Asterisks denote significant differences between genotypes at p < 0.05 (*), p < 0.01 (**) and p < 0.001 (***).

To understand the relationship between duration of photosynthesis in the flag leaf and grain filling, we compared grain filling parameters in *NAM* RNAi and control plants (Fig 2). In general, the two genotypes were very similar. They accumulated equivalent amounts of starch, to a maximum at 40 DAA (Fig 2A). Grain fresh weights were similar through development, except that *NAM* RNAi grains had higher fresh weights than control grains at 45 DAA (13% higher; Fig 2B). At 45 DAA moisture content in *NAM* RNAi grains was markedly higher than control grains (34% compared to 21%; Fig 2C) which contributed to the fresh weight difference (Fig 2B). At 30 and 35 DAA moisture content was also marginally higher in *NAM* RNAi grains (Fig 2C). The maximum dry weight, at 45 DAA, was the same in the two genotypes, although the dry weight of *NAM* RNAi grains was marginally less than that of control grains between 30 and 40 DAA (Fig 2D).

Grains of the two genotypes had similar sugar levels during most of development (Fig 2E, 2F and 2G). Sucrose was the major sugar, and levels changed relatively little during development. Levels of sucrose were the same in *NAM* RNAi and control grains up to 30 DAA, but were about 33% higher in *NAM* RNAi grains at 40 and 45 DAA. Hexose levels per grain fell during development. Glucose levels were the same in the two genotypes except at 45 DAA, when the level was two-fold higher in *NAM* RNAi grains than in control grains. Fructose levels were the same except at 20 DAA when *NAM* RNAi grains had one third less than control grains. The activities of the starch synthesising enzymes ADP glucose pyrophosphorylase (AGPase) and starch synthase increased up to about 20 DAA and then declined (Fig 2H and 2I). The enzyme activities were the same in the two genotypes except at 20 DAA when they were lower in *NAM* RNAi than control grains.

### Fructan is retained in stems of *NAM* RNAi plants

**Fig 3:**
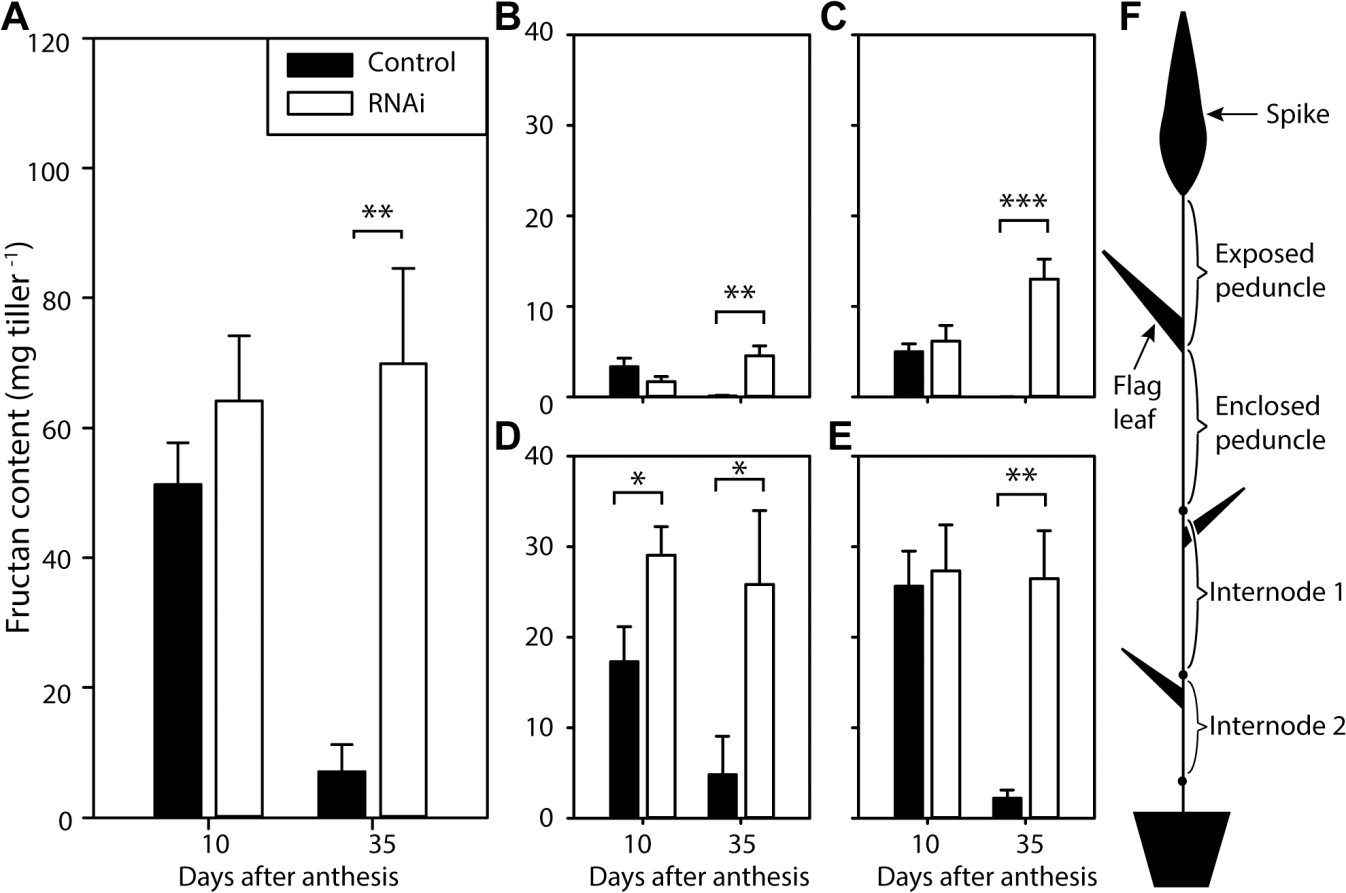
Fructan content of stem tissues in L19 *NAM* RNAi and control plants. (A) Peduncle and internode tissues combined, (B) exposed peduncle, (C) enclosed peduncle, (D) internode 1 and (E) internode 2, (F) tissues harvested. Values are means of measurements on six individual plants (biological replicates), ± SEM. Asterisks denote significant differences between genotypes at p < 0.05 (*), p < 0.01 (**) and p < 0.001 (***).

To understand the effects of delayed leaf senescence on whole plant carbohydrate status we examined the accumulation of fructan in the stems (Fig 3). At 10 DAA, stems of *NAM* RNAi and control plants contained similar amounts of fructan (Fig 3A). Fructan content decreased from 51 to 7 mg tiller^-1^ in control plants from 10 to 35 DAA (Fig 3A), but in *NAM* RNAi plants fructan content was similar at 10 and 35 DAA (~70 mg tiller^-1^ Fig 3A). Thus *NAM* RNAi stems contained 63 mg tiller^-1^ more fructan than those of control plants at 35 DAA, accounting for 13% of the 495 mg additional glucose equivalents assimilated per flag leaf during the prolonged period of photosynthesis.

The net remobilisation of fructan in control plants and retention of fructan in *NAM* RNAi plants occurred throughout the stem tissues, including the exposed peduncle, enclosed peduncle, internode 1 and internode 2 (Fig 3B, 3C, 3D, 3E and 3F). The internode tissues had the highest fructan contents at 10 DAA in both genotypes. At 35 DAA the largest difference between *NAM* RNAi and control plants was in the internodes, which contained >21 mg tiller^-1^ more fructan in *NAM* RNAi plants than in control plants (Fig 3D and 3E). The exposed and enclosed peduncle of *NAM* RNAi plants contained 4 and 13 mg tiller^-1^ more fructan than control plants, respectively (Fig 3B and 3C).

## Discussion

In this study we exploited two near-isogenic lines of wheat with very different timing of flag leaf senescence to study the impact of a prolonged period of flag leaf photosynthesis on grain filling. Although the flag leaves of *NAM* RNAi lines continued to photosynthesise at a maximum rate for ten days longer than those of control plants, the additional photosynthate did not result in a higher grain mass in the *NAM* RNAi lines.

### Starch synthesis capacity may limit grain filling

Our analysis of developing grain suggested that grain filling was limited at later stages by the capacity for starch synthesis rather than by substrate availability in both genotypes. First, the activity of starch synthase was comparable with the rate of starch synthesis thus it seems likely that starch synthase activity was limiting towards the end of grain filling. For example, grains accumulated about 5 mg starch between 30 and 40 DAA, equivalent to a rate of starch synthesis of approximately 2 nmol min^-1^ grain^-1^. The measurable activity of starch synthase was the same as or somewhat lower than this value in the same period. A second piece of evidence for starch synthesis capacity limiting grain filling is that sucrose levels per grain changed relatively little through development, implying that substrate availability was maintained even when the rate of starch synthesis was declining towards the end of grain filling. This was seen in both control and *NAM* RNAi plants. The fact that sucrose content was higher in *NAM* RNAi grains than in control grains at 40 DAA–when photosynthesis was continuing in *NAM* RNAi but not control flag leaves–and that *NAM* RNAi plants accumulated fructan in their stems, also supports the view that the cessation of starch synthesis in this genotype was due to limited capacity for grain filling.

Previous studies have also concluded that duration of grain filling may be limited by starch synthase activity. It is generally observed that this activity is comparable with the rate of starch synthesis, and declines sharply during the later stages of grain development [28–31]. Other enzymes on the sucrose-to-starch pathway are usually reported to have much higher activities than required to support the rate of starch synthesis, and transgenic wheat plants with higher rates of sucrose uptake into the grain did not have higher starch accumulation than control plants [32]. Attempts to measure the flux control coefficient of starch synthase in developing grain, by exploiting its extreme thermal instability to specifically reduce its activity, have also concluded that the rate of starch synthesis is controlled predominantly by starch synthase activity [33].

### Potential yield increase from extended flag leaf photosynthesis

The limitation imposed by starch synthase activity implies that prolongation of flag leaf photosynthesis alone is unlikely to increase grain mass. However given appropriate manipulation of sink capacity, prolongation of photosynthesis could potentially increase grain yield. To investigate the magnitude of this potential increase, we first estimated what fraction of carbon in the grain may have been derived directly from flag leaf photosynthesis in control plants. Each control plant produced approximately 8 g of grain (Table 1). Photosynthesis in the flag leaves of control plants produced about 5 g of glucose equivalents between 0 and 30 DAA (based on our estimate of 2 g glucose equivalents from 10 days of photosynthesis, and assuming that the average rate over 20 to 30 DAA was half the maximum rate). Assuming that 25% of the carbon entering the grain is respired [34] and hence that the production of 8 g of grain requires 10.7 g of carbon, photosynthesis in the flag leaves could thus account directly for up to 47% of the grain mass. All of this contribution would occur before 30 DAA, when photosynthesis ceased in control flag leaves. After about 20 DAA, carbon for grain filling presumably comes increasingly from remobilisation of materials in the senescing leaves, and from mobilisation of stem reserves [35, 36]. The remaining carbon could be provided by photosynthesis of stems, lower leaves and ears. Ear photosynthesis is estimated to contribute 10–60% of grain carbon, depending on cultivar, growth conditions and methods of measurement [37–40]. In *NAM* RNAi plants, photosynthesis in the flag leaf could potentially make a greater contribution to grain filling than in control plants, over a longer period. The prolonged period of flag leaf photosynthesis in *NAM* RNAi produced approximately 2 g more glucose equivalents per plant than was available in control plants. Using the assumptions above, this represents a potential increase in grain mass of about 20%. This is of course a maximum estimate since the fate of flag leaf photosynthate will be determined by competition between the grain and other sink organs.

### Carbohydrate metabolism in the context of micronutrient remobilisation

The *NAM* RNAi lines used in this study have been reported to not only have delayed senescence, but also to have reduced nutrient content in their grains [23, 41]. The similarity in carbohydrate accumulation during grain filling in *NAM* RNAi and control grains, is consistent with previous experiments which found that nitrogen (N), copper (Cu), iron (Fe) and zinc (Zn) accumulate to similar levels in the grain of both genotypes until 35 DAA (corresponding to maximum grain dry weight when starch accumulation has ceased, similar to the present study) [41]. From 35 DAA *NAM* RNAi grain accumulated less N, Cu, Fe and Zn than control grain, resulting in *NAM* RNAi grain containing 30% less of these nutrients at maturity than control grain [23, 41]. In vegetative tissues N, Cu, Fe and Zn contents were similar in *NAM* RNAi and control plants until 35 DAA but after this point *NAM* RNAi plants remobilised lower amounts of these nutrients than control plants. In contrast, we found that *NAM* RNAi and control plants differed strongly with respect to stem fructan remobilisation by 35 DAA, implying that this difference arose considerably earlier than the difference in mineral remobilisation. This difference in timing suggests that low remobilisation of fructan and of minerals in *NAM* RNAi plants are distinct phenomena. We suggest that failure to remobilise fructans is a direct consequence of the prolongation of flag leaf photosynthesis in *NAM* RNAi plants, whereas the later failure of mineral remobilisation may be linked to the late timing of flag leaf senescence in these plants.

### Future directions

Our results are consistent with previous studies which suggest that photosynthate availability does not limit grain filling, and hence that efforts to increase grain filling capacity will be important in achieving higher yields in wheat. An obvious approach would be to increase capacity for starch synthesis–in particular the activity of starch synthase—in the latter part of grain development. Transcript levels for starch synthases and other enzymes of starch synthesis start to decline early in grain development, when the rate of starch accumulation is high [42–46], hence it may be possible to prolong the period of starch synthesis by extending the period over which genes encoding the relevant enzymes are expressed.

In addition to making better use of available photosynthate, an increase in grain filling capacity may also increase the rate of photosynthesis, with potentially synergistic effects on yield [13]. There is evidence that photosynthesis during grain filling may be suppressed by end-product inhibition. Rates of photosynthesis are reduced by treatments that limit the export of assimilate from leaves or reduce sink strength (for example by grain removal) and hence assimilate utilization [47–49]. Conversely, removal of some leaf material during grain filling can increase the rate of photosynthesis in remaining leaves [50]. Thus manipulation of grain filling and photosynthetic capacity in tandem presents an as yet untapped opportunity for significant increases in wheat yield.

## Supporting Information

S1 Fig Flag leaf senescence characteristics of L23 *NAM* RNAi and control plants. Measurements were made exactly as for L19 plants, as described in Fig 1. A) Chlorophyll. Values are means of the average measurements for flag leaves from six plants (biological replicates), ± SEM (standard error of the mean). B) Photosynthesis. Values are means of measurements on six plants (biological replicates), ± SEM. Asterisks denote significant differences between genotypes at p < 0.05 (*).
(TIF)

S1 Table Architecture and yield components of L23 *NAM* RNAi and control plants. (DOCX)
