# Supplementary figures and images for: Wheat Grain Filling Is Limited by Grain Filling Capacity rather than the Duration of Flag Leaf Photosynthesis: A Case Study Using NAM RNAi Plants
Source: PLoS One. 2015 Aug 4;10(8):e0134947. doi: 10.1371/journal.pone.0134947 (PMC4524614; doi:10.1371/journal.pone.0134947)

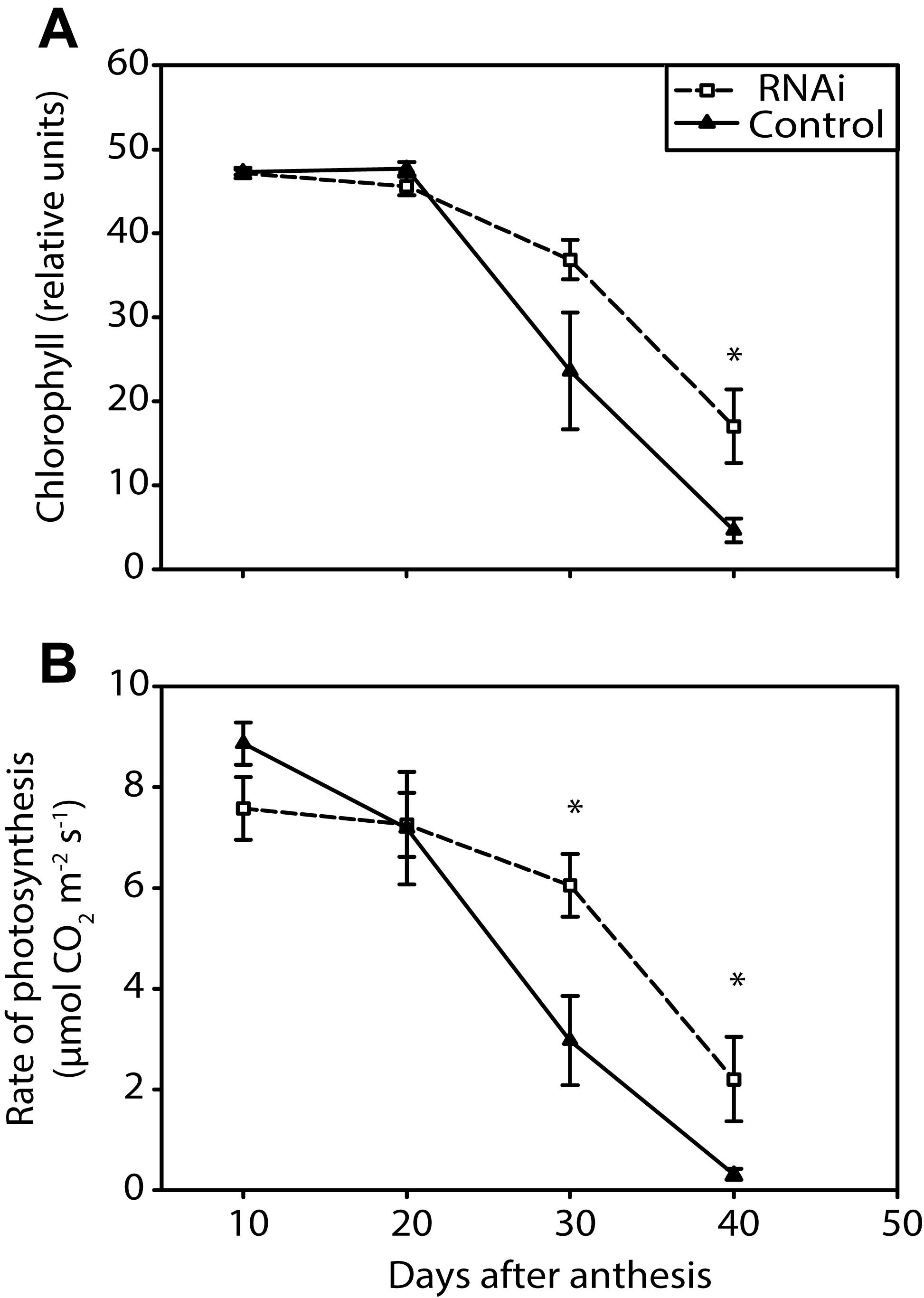

Supplement: S1 Fig — Measurements were made exactly as for L19 plants, as described in Fig 1. A) Chlorophyll. Values are means of the average measurements for flag leaves from six plants (biological replicates), ± SEM (standard error of the mean). B) Photosynthesis. Values are means of measurements on six plants (biological replicates), ± SEM. Asterisks denote significant differences between genotypes at p < 0.05 (*). (TIF) [file pone.0134947.s001.tif]
