# Supplementary material for: Wheat Grain Filling Is Limited by Grain Filling Capacity rather than the Duration of Flag Leaf Photosynthesis: A Case Study Using NAM RNAi Plants
Source: PLoS One. 2015 Aug 4;10(8):e0134947. doi: 10.1371/journal.pone.0134947 (PMC4524614; doi:10.1371/journal.pone.0134947)
Supplement: S1 Table — (DOCX) [file pone.0134947.s002.docx]

**S1 Table. Architecture and yield components of L23 *NAM* RNAi and control plants.**

|  | **Control** | **RNAi** |
| --- | --- | --- |
| Main tiller flag leaf area (cm^2^) | 39.3±1.7 | 39.6±1.7 |
| Tillers per plant | 4.0±0.3 | 4.0±0.3 |
| Grain mass per plant (g)^b^ | 5.7±0.25 | 4.0±0.3 |
| Thousand grain weight (g)^b^ | 37.1±0.6 | 32.8±0.7 |
| Grain number per plant^a^ | 147.7±7.9 | 117.0±8.8 |
| Grain area (mm^2^) | 17.5±0.25 | 16.6±0.17 |
| Grain length (mm)^a^ | 5.9±0.03 | 5.8±0.04 |
| Grain width (mm) | 3.5±0.03 | 3.5±0.02 |

Values are means of measurements on 12 individual plants (biological replicates), ± SEM (standard error of the mean). Grain measurements include grains from all tillers.

^a^ Significant difference between genotypes at p < 0.05 (*).^b^ Significant difference between genotypes at p < 0.001 (***). Other parameters were not significantly different between genotypes.
